# Risks in Management of Enteral Nutrition in Intensive Care Units: A Literature Review and Narrative Synthesis

**DOI:** 10.3390/nu13010082

**Published:** 2020-12-29

**Authors:** Magdalena Hoffmann, Christine Maria Schwarz, Stefan Fürst, Christina Starchl, Elisabeth Lobmeyr, Gerald Sendlhofer, Marie-Madlen Jeitziner

**Affiliations:** 1 Executive Department for Quality and Risk Management, University Hospital Graz, 8036 Graz, Austria; magdalena.hoffmann@medunigraz.at (M.H.); gerald.sendlhofer@medunigraz.at (G.S.); 2 Research Unit for Safety in Health, c/o Division of Plastic, Aesthetic and Reconstructive Surgery, Department of Surgery, Medical University of Graz, Auenbruggerplatz 1/3, 8036 Graz, Austria; 3 Division of Endocrinology and Diabetology, Department of Internal Medicine, Medical University of Graz, 8036 Graz, Austria; christina.starchl@stud.medunigraz.at; 4 Division of Gastroenterology and Hepatology, Department of Internal Medicine, Medical University of Graz, 8036 Graz, Austria; stefan.fuerst@medunigraz.at; 5 Intensive Care Unit 13i2, Department of Medicine I, Medical University of Vienna, 1090 Wien, Austria; elisabeth.lobmeyr@meduniwien.ac.at; 6 Department of Intensive Care Medicine, University Hospital Bern, Inselspital, University of Bern, 3010 Bern, Switzerland; Marie-Madlen.Jeitziner@insel.ch

**Keywords:** patient safety, enteral nutrition, intensive care unit, quality, risk management

## Abstract

Critically ill patients in the intensive care unit (ICU) have a high risk of developing malnutrition, and this is associated with poorer clinical outcomes. In clinical practice, nutrition, including enteral nutrition (EN), is often not prioritized. Resulting from this, risks and safety issues for patients and healthcare professionals can emerge. The aim of this literature review, inspired by the Rapid Review Guidebook by Dobbins, 2017, was to identify risks and safety issues for patient safety in the management of EN in critically ill patients in the ICU. Three databases were used to identify studies between 2009 and 2020. We assessed 3495 studies for eligibility and included 62 in our narrative synthesis. Several risks and problems were identified: No use of clinical assessment or screening nutrition assessment, inadequate tube management, missing energy target, missing a nutritionist, bad hygiene and handling, wrong time management and speed, nutritional interruptions, wrong body position, gastrointestinal complication and infections, missing or not using guidelines, understaffing, and lack of education. Raising awareness of these risks is a central aspect in patient safety in ICU. Clinical experts can use a checklist with 12 identified top risks and the recommendations drawn up to carry out their own risk analysis in clinical practice.

## 1. Introduction

In intensive care units (ICUs) critically ill patients have a high risk of developing malnutrition which is associated with a poorer clinical outcome [1]. Therefore, enteral nutrition (EN) has become an increasingly important research topic in recent years. In 2017, Arabi et al. (2017) reported that the intensive care medicine research agenda in nutrition and metabolism includes topics like optimal protein dose combined with standardized active and passive mobilization during the acute and post-acute phases of critical illness, nutritional assessment and nutritional strategies in critically obese patients. Moreover, the effects of continuous versus intermittent EN were classified as a hot topic [2]. The European Society for Clinical Nutrition and Metabolism (ESPEN), established standard operating procedures (SOPs) and guidelines for the provision of the best nutritional therapy for critically ill patients. They are regularly updated and cover various aspects of medical nutritional therapy such as duration, timing, vulnerable patient populations with, for example, dysphagia or frailty and provide clinicians with practical procedures [3]. However, in clinical practice, EN is often not given highest priority due to other symptomatic problems such as cardiovascular status or the need of ventilation. Moreover, ICU patients are mostly very heterogeneous in terms of their illness, resulting in multiple risks and safety issues for patients. The literature offers possible solutions to this problem, but many unresolved questions still cannot be answered conclusively. However, there is some degree of consensus. For ventilated patients in ICUs, if possible, it is crucial to prefer EN over parenteral nutrition (PN) [3]. Other topics like the microbiome or recommendations on additives such as micronutrients and vitamins are still being discussed, for example, optimal vitamin D levels. Many critically ill patients suffer from vitamin D deficiency (serum 25-hydroxyvitamin D [25(OH)D] < 20 ng/mL), with levels lower than 12 ng/dL [4].

In addition to the clinical impact there are further uncertainties that affect the outcome of patients. Overall, human errors are the third leading cause why patients die in a hospital [5]. ICUs are in general a high-risk area, where critical ill patients receive a highly sophisticated care. Patients receive a lot of drugs; medical devices are used for administration of drugs and for ventilation. To increase patient safety, clinical risk management focuses on improving the quality and safety of health care services by identifying the circumstances and opportunities that expose patients to risk of harm, and on acting to prevent or control those risks [6]. In fact, clinical risk management is a key element of clinical governance and management [7]. Risks must be handled appropriately with a bundle of measures and they need to be audited regularly to ascertain whether risk-reducing measures are being used sufficiently [8]. If risks are unknown, a risk management audit is needed to identify potential risks. Especially in EN, there are several risks such as a failure in reaching the nutrition target (CIRSmedical^®^ no. 26 (V1)). The aforementioned error can lead to hypoglycemia and malnutrition [9]. Furthermore, EN and aspiration [10], which can occur when a patient was not placed in a head-up position or when post-pyloric feeding (nasojejunal tube) was not administered to patients with a high risk of aspiration [11]. On top, the worldwide introduction of the DIN EN ISO 80369-3 and the use of ENFit™-technology was a decisive step to increase patient safety in EN and can be seen as an important contribution in terms of risk management. However, the implementation of this standard still has shortcomings as not all security gaps were eliminated. It is still possible that enteral medication in Luer injections or tube feeding can be administered intravenously [12]. In addition to ICU-related risks there are further general risks such as an incorrect patient identification, nosocomial infections, medication errors, overlooked allergies, insufficient pain management, and failures in communication and documentation or failures in handling medical devices [6]. A consequent risk management in ICUs can help to increase patient safety; therefore, the aim of this rapid literature review was to identify risks for patient safety in the management and handling of EN in critically ill patients in ICU.

## 2. Materials and Methods

We conducted a literature review guided by the Rapid Review Guidebook by Dobbins (2017) [13] and the PRISMA Checklist [14]. As the first step in the EN risk identification process, we (MH, CMS, SF, CS, EL) used feedback from ICU staff (physicians, nurses, dieticians), reports from the Critical Incident Reporting System (CIRS) of the University Hospital of Graz, Austria, CIRSmedical^®^ and, online, the “berrypicking” method [15]. We structured these risks in a process: admission, prescription, verification, preparation, administration, monitoring, discharge, and general risks in EN in the ICU. We then systematically identified and screened the literature for these risks.

### Search Strategy

Keywords, related MeSH terms and Boolean operators (AND, OR) were used. The main terms in the first search were: enteral nutrition, risks and intensive care and the identified risks. For example, “enteral nutrition” AND “risks” OR safety AND “intensive care unit” AND “aspiration”. At least two different terms were used for each risk. The primary search was limited to systematic reviews, meta-analyses, and guidelines. Next, studies, including randomized controlled trials (RCTs), retrospective studies and prospective observational studies were included to detect possible risks in EN in the ICU. The search was done with a language restriction to German and English and available full-text articles. Three different scientific databases, PubMed, Cochrane Library and Web of Science, were used to identify studies between 2009 and 2020.

Four independent reviewers (CMS, SF, CS, EL) searched in scientific databases between June and August 2020. The search was always carried out by two independent researchers in order to reduce a possible selection bias. The risks and the related literature were reviewed by independent researchers (CMS, SF, CS, EL and senior researcher (MMJ) with research experience >10 years). After the decision for inclusion of studies, the results were viewed and discussed by two researchers (MH, CMS). All identified risks were thematically summarized in a narrative synthesis by one senior researcher (MMJ) and checked by another researcher (MH). Data were prepared for a summary in a narrative synthesis and a checklist.

## 3. Results

In total we found 20 risks. We included 12 risks in our top risk list, which we could verify in the literature. We had 3495 hits in scientific databases (PubMed *n* = 1301, Web of Science *n* = 2102, Cochrane *n* = 92) and found 146 relevant studies and included 62 in our narrative synthesis. A detailed presentation of the included literature can be found in the following Table 1.

**Figure 1:**
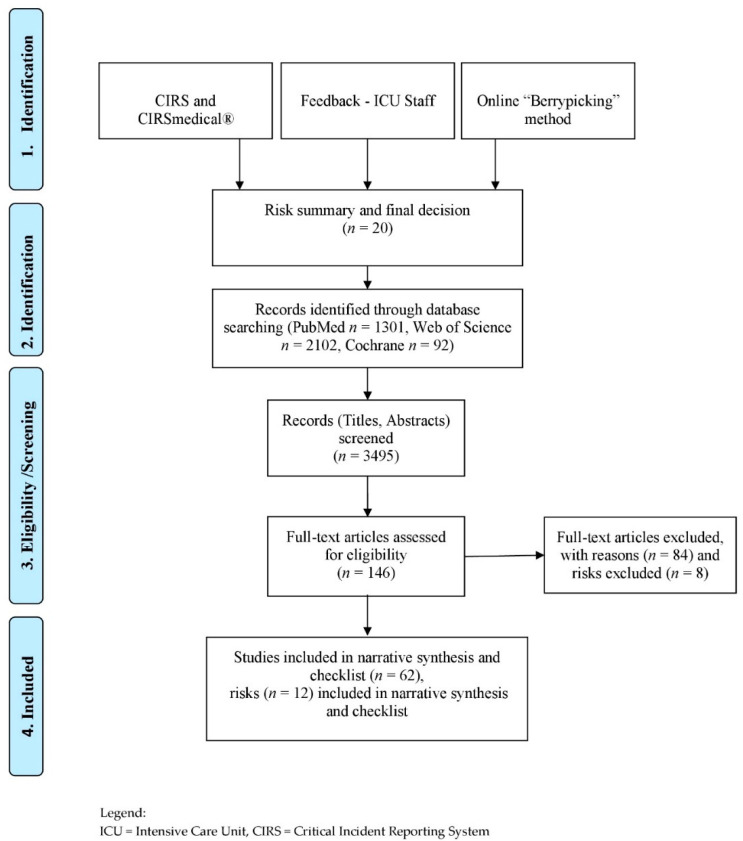
Flow Diagram—Risk identification process and narrative synthesis.

The identified studies focused primarily on assessments or interventions and less on risks or safety issues. These risks and safety issues were derived from the studies directly and indirectly. A detailed description of the search process can be found in Figure 1 (by Moher et al. (2009) [14] adapted by Hoffmann).

### 3.1. Admission

#### 3.1.1. No Use of Clinical Assessment or Screening Nutrition Assessment

Singer et al. (2019) primarily recommended a general clinical assessment, including a history, report of weight loss or decrease in physical performance before ICU admission, physical examination, etc., in addition to screening and assessment instruments. Presence of frailty is considered to be significant in ICU patients, and should therefore be considered in nutritional management. Recording of muscle mass is also used as a parameter for assessing the nutritional status [3]. In a systematic review, Lew et al. (2017) investigated nutrition assessment tools such as the Subjective Global Assessment (SAG) and Mini Nutritional Assessment (MNA) on the one hand and, on the other hand, nutrition screening instruments like the Nutrition Risk Screening 2002 (NRS-2002) and the Malnutrition Universal Screening Tool (MUST) [1]. Further studies focused on instruments such as the NUTRIC score and the mNUTRIC score [16,17,18,19,20,21]. Given that the studies investigated different illnesses, different instruments, various concepts such as nutrition screening or assessment, and that there is no clear definition of critical illness-associated malnutrition [3], various risks can arise in practice. However, studies and guidelines recommended a clinical assessment supplemented by an easy-to-use validated screening instrument such as the NRS-2002 (NRS-2002) and the NUTRIC score—determine both nutrition status and disease severity, while the use of a frailty scale can be helpful for elderly patients.

#### 3.1.2. Inadequate Tube Management and Position

Singer et al. (2019) recommended the use of gastric access as the standard approach to initiate EN. Post-pyloric, mainly jejunal, feeding is possible for patients deemed to be at high risk of aspiration [3]. Ultrasonography, camera-assisted technology with real-time video guidance and X-rays are used to check the position of the tube after insertion and thus obtain a positive outcome [22,23,24]. In clinical practice further tube placement testing methods are used such as aspirate appearance, aspirate pH or auscultation, although X-rays and real-time video guidance are considered to be the most adequate methods. Different types of tubes designed for use with imaging procedures should also be considered. However, certain questions remain unanswered with regard to risk management, for example how long the tubes remain in the correct position, or after what period of time or type of intervention a further positional check is needed, including for jejunal tubes. It is important for individual ICUs to develop a protocol to guide their tube management policy.

### 3.2. Prescription

#### Missing Energy Target

For mechanically ventilated patients, guidelines and studies recommended that EN should be determined by indirect calorimetry [25,26,27]. In the absence of indirect calorimetry, VO2, or VCO2 measurements and simple weight-based equations (such as 20–25 kcal/kg/d) should be used. In order to prevent risks, it is important that the prescribed quantity should match the calorie requirement, and that this should be re-evaluated in regular intervals [28], as the measured energy expenditure increased in the course of time with great individual variation [29]. De Waele et al. (2012) recommended a dedicated nutrition support team for a more systematic use of indirect calorimetry in long-term mechanically ventilated patients [30]. However, in order to determine patients’ energy target, their nutritional status before admission to ICU should not be used [3].

### 3.3. Verification

#### Missing a Nutritionist at the ICU

Two studies, examining the clinical impact of a two-step interdisciplinary nutrition program and enteral feeding protocols in the ICU, have found that interventions by a dietician significantly improved patient energy balances by day 7 [31,32]. Additionally, the presence of a dietician in the ICU has been associated with better nutrition performance, with a multi-professional approach reducing risks through shared responsibility, interdisciplinary quality programs, and re-evaluations of EN [33]. A systematic review by Mistiaen et al. (2020), stated, that there is weak evidence that Nutrition Support Teams increase appropriate EN use in ICU patients. A decrease of the duration of PN could not be shown [34].

### 3.4. Preparation

#### Insufficient Hygiene and Handling

In their study, Perry et al. (2015) compared open systems, “ready-to-hang”-systems (RTH), and modular hospital-built tube feeding systems (MTF), in a normothermic (23 °C) and hypothermal ICU environment. The contamination in both environments/systems does not differentiate between open and closed feeding systems for up to 8 h. However, adding modules to open systems can lead to an unacceptable risk of contamination in hyperthermic (i.e., particularly warm) environments [35]. Training in preparing the setups, maintaining constant temperatures before and after preparation, as well as storage were important factors.

### 3.5. Administering

#### 3.5.1. Wrong Time Management, Speed and Route

Medical nutrition therapy should be considered for all ICU patients, mainly for those staying for more than 48 h [3]. An oral diet is preferable to EN or PN for critically ill patients who are able to eat. If oral intake is not possible, early EN (within 48 h) in critically ill adult patients should be performed/initiated without delay [3,36,37,38]. To avoid overfeeding, early full EN and PN should not be used in critically ill patients but should be prescribed within three to seven days. Singer et al. (2019) suggest to be caution in critically ill patients with uncontrolled shock, uncontrolled hypoxemia and acidosis, uncontrolled upper gastrointestinal bleeding, gastric aspirate >500 mL/6 h, bowel ischemia, bowel obstruction, abdominal compartment syndrome, and high-output fistula without distal feeding access. A systematic review and a meta-analysis showed that, when given within 48 h after admission, EN itself is efficient and safe for those patients with predicted severe acute pancreatitis [39]. An episode of vomiting was observed in patients with sepsis [40]. Blaser et al. (2017) and Zheng et al. (2019) confirmed that early EN reduced infectious complications in unselected critically ill patients, and in traumatic brain injury, severe acute pancreatitis, gastrointestinal (GI) surgery and abdominal trauma [41,42]. However, their recommendations are weak because of the poor quality of the evidence, with several information based only on expert opinion. In order to actively counter the risks, early EN should be monitored like a vital sign. Though the implementation of the ENFit™-standard it is still possible that enteral medication in Luer injections or tube feeding can be administered intravenously. A systematic review and meta-analysis by Alkhawaja et al. (2015) evaluated the effectiveness and safety of post-pyloric feeding versus gastric feeding. There was no difference in mortality or duration of mechanical ventilation but post-pyloric feeding is associated with lower rates of pneumonia compared with gastric tube feeding [43].

#### 3.5.2. Nutritional Interruptions

Lee et al. (2018) reported 332 episodes of feeding interruptions, this means 12.8% (4190 h) of the total 1367 nutrition days. Each ICU patient experienced feeding interruptions for a median of three days. Total duration of feeding interruptions for the entire ICU stay: 24.5 h, which resulted in an energy and protein deficit. They therefore recommended an evidence-based feeding protocol and a nutrition support team [44]. Williams et al. (2013) investigated the number of nutritional interruptions. They cited education, audit, leadership support, interprofessional collaboration and the use of guidelines as starting points for reducing these interruptions [45]. Based on a chart review, Uozumi et al. (2017) also proposed the development of a protocol for nutritional interruptions, this could possibly reveal deficits in the administration of the EN at an early stage [46]. Prolonged fasting before and after surgery, airway procedures, dressing changes, feed intolerance, and tube malfunction were identified as the most important causes of delays by Segaran et al. (2016) [47,48].

#### 3.5.3. Wrong Body Position

To reduce gastric residual volume in ICU patients, Farsi et al. (2020) recommended positioning patients in the right lateral and supine, semi recumbent positions rather than in the supine position [49]; however, this remains contradictory. There is insufficient literature on this subject. The use of a protocol based on the elevation of the patient’s head, the use of fixed prokinetics and reduced speed of the diet allowed the application of early EN and faster attainment of the planned energy target in prone position, this was found by Regnier et al. (2009) [50]. However, the literature regarding the effect of EN while in the prone position is also sparse and of limited quality [51]. No studies on agitated patients, whose position can change continuously, have been found. Therefore, to prevent risks, ongoing clinical observations are needed.

### 3.6. Monitoring

#### Gastrointestinal Complication and Infections

Digestive complications in EN were found. Most often described as vomiting, diarrhea, bowel ischemia, and acute colonic pseudo-obstruction [52,53,54,55,56] these complications were considered as risk factors for extended ICU stay and prolonged mechanical ventilation [55,56,57]. Further complications included refeeding hypophosphatemia and aspiration [11,58,59,60]. However, comparing early EN versus early PN, Singer et al. (2019) showed in their results, a reduction of infectious complications, shorter ICU and hospital stay in EN. They recommended the use of EN over PN in patients with an intact gastrointestinal tract.

### 3.7. General Risk

#### 3.7.1. Missing or Not Using Existing Guidelines, Standards or Protocols

Singer et al. (2019) recommended the implementation of evidence based protocols [3]. Studies showed significant improvement in EN delivery and reduced duration of feed breaks when using a protocol [39,61,62]. EN is even started earlier [32]. The main barriers to EN guideline compliance were delays, unpredictable timing of procedures, and differing guidance from senior staff and non-ICU teams [3,32,61,63].

#### 3.7.2. Understaffing

Risk factors for inadequate nutrition support as described by Honda et al. (2013), Darawad et al. (2018), Cahill et al., (2012), and Huang et al., (2019) included fewer nursing professionals per bed, and a lack of dietitian coverage during weekends and holidays [42,64,65,66,67].

#### 3.7.3. Lack of Education

Studies show that multifaceted nutritional education programs and protocols are able to improve the knowledge of the healthcare professionals. These tools and training programs should be versatile, easy to use, and can be web-based [68,69,70,71,72,73,74,75].

## 4. Discussion

Our narrative synthesis highlights the risks of EN in ICU. Each process step, from admission to discharge, demonstrated certain risks, like no use of clinical assessment or screening nutrition tools, inadequate tube management and position, missing energy target, missing a nutritionist at the ICU, bad hygiene and handling, wrong time management and speed, nutritional interruptions, wrong body position, gastrointestinal complication and infections, missing or not using guidelines, standards or protocols, understaffing, and lack of education which are often intertwined and mutually dependent. Due to the heterogeneity of the ICU patient population, no consensual evidence-based protocols about EN were found. However, studies indicated, that for all the aforementioned risks, safety measures exist.

In health care systems in recent years, patient safety has become a priority issue [6]. In addition to individual measures, national and international strategies and protocols have attempted to overcome the most prominent hazards. In clinical risk management it is important to identify, analyze, and manage potential risks. The implementation of measures into routine procedures within complex hospital organizations like ICUs is challenging and have to be monitored regularly as adherence or compliance can be lacking [8].

Based on our findings we developed a short checklist (see Table S1) which can be used by key groups like ICU staff to detect risks in ICUs. Diverse reviewers, with different years of work experience, should use the checklist independently (e.g., nurses, physicians, and dieticians), because a person’s knowledge is not the department’s knowledge. Application of the checklist should be followed by a discussion of the results, implementation of relevant measures and ways of how a distinctive measure can be checked in the routine.

In our results, we did not describe all identified risk which were reported by staff and CIRS, such as interaction between medication or other additives and EN, involvement of relatives and missing discharge plans. This does not imply that these risks and safety issues do not exist, but reflects the lack of adequate literature. Due to a very high turnover in ICU staff [76] and a very complex setting, the implementation of multifaceted nutritional education programs and protocols based on the newest guidelines [3] are necessary to improve and secure the knowledge of healthcare professionals. These tools and training programs should be versatile, easy to use, can be web based [68,69,70,71,72,73,74,75] and trained at regular intervals, followed by internal and external audits [8].

### Strengths and Limitations

Our narrative synthesis highlighted risks of EN in ICU. These risks can be observed, and institutional approaches exist for minimizing these risks, for example, by raising awareness, evidence-based protocols, guidelines, consulting professionals, and education. The narrative synthesis should prompt the readers to reflect on their own way of working and on the actual and potential risks. However, our study has several limitations. First, due to the lack of systematic reviews, we also included other studies with different methods and missing high-quality evidence. Depending on the study type, bias is possible. Many of these previous nutrition trials were open to bias because they were unblinded, very small or had other confounders. Second, our literature languages were restricted to German and English and the search terms were also limited due to a large number of studies. Published guideline recommendations for the management of nutrition in ICU patients remain largely supported by expert opinion and only a minority of the studies and reports includes high-quality evidence [77]. Finally, few of the identified studies addressed the risks and safety issues of EN directly, therefore further research is needed.

## 5. Conclusions

The aim was to identify risks in the management of EN in critically ill patients in ICU. Based on our results, numerous risks related to the management of EN in the ICU were discovered. Clinical experts can use the risk checklist and the recommendations drawn up to carry out their own risk analysis in clinical practice. Once risks have been identified, appropriate measures can be taken. From the authors’ point of view, risk management with tools such as checklists or other risk analysis tools are important for improving patient safety in the ICU and secure knowledge. Further research is needed on how risk management can be implemented in the daily routine, so that the staff reflects on and reviews their own EN management.

## Figures and Tables

**Table 1:** Literature, terms, and hits.

**Country**	**Study Design**	**Objective of Study/Outcomes**	**Study Population/Setting**	**Results/Consequence(s)**	**Reference**
Israel	Guideline	ESPEN Guideline “Clinical Nutrition on intensive care unit”	Literature review and expert opinion	Possible complications as well as energy and protein deficit.	[1]
Australia	Systematic review	To determine whether malnutrition diagnosed by validated nutrition assessment tools such as the Subjective Global Assessment (SGA) or Mini Nutritional Assessment (MNA) is independently associated with clinical outcomes and if the use of nutrition screening tools demonstrate a similar association.	20 case-control or cohort studies, adults in the ICU; Outcomes including mortality, length of stay (LOS), incidence of infection (IOI).	Prevalence of malnutrition ranged from 38% to 78%. Malnutrition diagnosed by nutrition assessments was independently associated with increased ICU LOS, ICU readmission, IOI, and the risk of hospital mortality. The SGA had a better predictive validity than the MNA. Compared with nutrition assessment tools the predictive validity of nutrition screening tools was less consistent.	[2]
Canada	Post hoc analysis of an existing database derived from a RCT.	To externally validate a modified version of the NUTRIC score.	1199 ICU patients with multi-organ failure, mechanically ventilated, with expected length of stay >5 days with a primary outcome of 28-day mortality.	Increased nutritional adequacy is associated with increased survival in patients with higher NUTRIC scores (>6) but not in patients with lower NUTRIC scores (<5). There is a strong positive association between nutritional adequacy and 28-day survival in patients with a high NUTRIC score but this association diminishes with decreasing NUTRIC score. Higher NUTRIC scores are significantly associated with higher 6-month mortality (*p* < 0.0001) and the positive association between nutritional adequacy and 6 months survival was significantly stronger in patients with higher NUTRIC score (*p* = 0.038).	[3]
Australia	Retrospective audit of patients	To evaluate the applicability of a nutrition triage tool against the NUTRIC score.	Patients (*n* = 151)	The NUTRIC score identified 18 positive responses. (18/49) in the general ICU population and 24 positive responses (24/102) in the cardiac surgical ICU population. Of these positive NUTRIC responses the current nutrition triage tool identified 83% (15/18) and 71% (17/24), respectively. The current tool also identified 39 patients (26%), across both subpopulations which were not identified by the NUTRIC score.	[4]
China	Prospective observational study, single center	Evaluation of nutrition risk assessment and prognosis with prediction tools (NRS-2002, NUTRIC, and mNUTRIC scores) in neurological ICU (NICU) patients. Primary outcome: 28-day-mortality.	140 critically ill neurological patients.	Based on their mNUTRIC scores, a high nutrition risk (mNUTRIC score ≥ 5) was observed in 28.6% of patients, whereas a low nutrition risk (mNUTRIC score < 5) existed in 71.4% of patients. NUTRIC and mNUTRIC score are both able to predict 28-day-mortality. Odds ratios of 3.30 (95% CI, 1.01–10.80), 3.54 (95% CI, 1.04–12.02), 10.88 (95% CI, 3.33–35.57), and 13.95 (95% CI, 4.32–45.01), respectively.	[5]
Australia	Retrospective observational study, single-center	To assess the feasibility of using the mNUTRIC tool to screen for patients at increased nutrition risk and to determine the proportion of those high-risk patients who were reviewed by a dietitian.	260 critically ill patients.	The median time required to complete a full mNUTRIC screen was 4 min and 54 s. During the study period, 160 patients admitted to the ICU were screened as being at low nutrition risk (mNUTRIC < 5). Of these patients, 63% (*n* = 101) were not reviewed formally by a dietitian. Eighty-one patients were flagged as high nutrition risk (mNUTRIC < 6); of these, 45 (56%) were formally reviewed by a dietitian and 36 (44%) did not have a dietetic consultation during their ICU stay.	[6]
USA	Retrospective analysis, single-center	To compare the Nutrition Risk in Critically Ill (NUTRIC) to the Nutritional Risk Screening (NRS) 2002 in terms of their associations with macronutrient deficit in ICU patients	312 adult critically ill patients	Mean NUTRIC and NRS 2002 scores were 4 ± 2 and 4 ± 1, respectively. Linear regression demonstrated that each increment in NUTRIC score was associated with a 49 g higher protein deficit (β = 48.70: 95% confidence interval [CI] 29.23–68.17) and a 752 kcal higher caloric deficit (β = 751.95; 95% CI 447.80–1056.09). Logistic regression demonstrated that NUTRIC scores > 4 had over twice the odds of protein deficits ≥ 300 g (odds ratio (OR) 2.35; 95% CI 1.43–3.85) and caloric deficits ≥ 6000 kcal (OR 2.73; 95% CI 1.66–4.50) compared with NUTRIC scores ≥ 4. No association of NRS 2002 scores with macronutrient deficit was observed.	[7]
Brazil	Retrospective cohort study, multicenter	To compare the nutrition risks detected by NUTRIC with those by NRS 2002 in order to identify whether both tools are concordant for equivalent use in clinical practice in the ICU.	208 critically ill patients	The comparison between both nutrition screening tools showed fair agreement (κ = 0.39). Nearly half of the patients were classified at high nutrition risk by NUTRIC (47.6%), whereas only approximately one-third of the sample was classified similarly by NRS 2002 (35.6%).	[8]
USA	Single center study, prospective case series	To identify the esophagus and the stomach, tube positioning via real-time video guidance with integrated real-time imaging system (*IRIS*).	49 critically ill patients hospitalized in the ICU or step-down unit, minimal feeding for 72-hours, 42 completed study.	After training, the tube placement was simple and safe. 44 subjects (92%) successfully placed. Median time 8 minutes till placement.	[9]
China	Single center study, case series	Enteral nutrition support to critically ill patients via the nasogastrojejunal approach guided by semi-automated ultrasound.	41 critically ill patients	The application of saline can be taken as sound window and the metal wire as the tracking target, the bedside nasogastrojejunal tube guided by semi-automated ultrasound is an effective feeding tube placement method. The total nursing service satisfaction of patients was 90.24%, and the total incidence of adverse reactions was 17.07%.	[10]
China	Single-center, randomized controlled trial	To compare the effectiveness of EM (electromagnetic)-guided and endoscopic nasoenteral feeding tube placement among critically ill patients. The primary end point was the total success rate of correct jejunal placement.	161 adult patients admitted to ICUs requiring nasoenteral feeding. Patients were randomly assigned to EM-guided or endoscopic nasoenteral feeding tube placement (1:1)	Success rate was achieved in 74/81 and 76/80 patients who underwent EM-guided and endoscopic jejunal tube placements, respectively (91.4% vs. 95%; relative risk, 0.556; (CI), 0.156–1.980; *p* = 0.360). The EM-guided group had more placement attempts, longer placement time, and shorter inserted nasal intestinal tube length. They had shorter total placement procedure duration and physician’s order–tube placement and order–start of feeding intervals. The EM-guided group had higher discomfort level and recommendation scores and lesser patient costs.	[11]
Israel	Pilot RCT (TICACOS), single center	To determine whether nutritional support guided by repeated measurements of resting energy requirements improves the outcome.	Mechanically ventilated patients (*n* = 130) Patients were randomized to receive enteral nutrition (EN) with an energy target determined either by indirect calorimetry measurements (study group, *n* = 56), or according to 25 kcal/kg/day (control group, *n* = 56).	The study group had a higher mean energy (2086 ± 460 vs. 1480 ± 356 kcal/day, *p* = 0.01) and protein intake (76 ± 16 vs. 53 ± 16 g/day, *p* = 0.01). There was a trend towards an improved hospital mortality in the intention to treat group (21/65 patients, 32.3% vs. 31/65 patients, 47.7%, *p* = 0.058) whereas length of ventilation (16.1 ± 14.7 vs. 10.5 ± 8.3 days, *p* = 0.03) and ICU stay (17.2 ± 14.6 vs. 11.7 ± 8.4, *p* = 0.04) were increased. A Kaplan–Meier curve for the ‘‘per protocol’’ group shows that hospital mortality was significantly lower in the study group (16/56 patients, 28.5% vs. 27/56 patients, 48.2%; *p* = 0.023. Survival at 60 days was 57.9 ± 9.9% in the study group and 48.1 ± 7.6% in the control group (*p* = 0.023).	[12]
Italy	Prospective, multicenter observational study	To evaluate the validity of predictive formulas and equations for the calculation of energy expenditure and protein needs, by using indirect calorimetry (IC) and the protein catabolite rate; and to compare prescribed and actually received nutrients with estimated and measured needs.	42 critically ill adult patients hospitalized with acute kidney injury.	There were 654 days of artificial nutrition. Average energy and protein prescribed were respectively 1551 ± 644 kcal and 70.5 ± 38.2 g, while energy and protein actually delivered were 1408 ± 651 kcal and 63.4 ± 35.3 g (*p* < 0.0001 for both comparisons). In general, average energy needs measured by IC were significantly higher than both the prescribed and delivered nutrient amounts (IC 1724 kcal ± 431; prescribed 1575 ± 672; received 1439 ± 680, *p* < 0.0001). No predictive formula was precise enough, and Bland–Altman plots wide limits of agreement for all equations highlight the potential to under- or overfeed individual patients.	[13]
Belgium	Prospective quality control study, single center	Whether feeding prescriptions were translated into adequate caloric intake within the scope of a “real-life,” guideline-oriented nutritional approach.	50 patients older than 18 years admitted to ICU when intubated and expected to receive mechanical ventilation for at least seven days.	24.6% of the 350 nutritional prescriptions correctly estimated the need. In 40.0% of cases, nutritional needs were insufficiently covered. Overestimation occurred in the remaining 35.4%. Caloric prescription resulted in accurate delivery in 56.0% of cases. Effective feeding was not met in 32.6% of prescriptions, and in 9.14% actual feeding surpassed the prescribed amount by more than 10%. This study demonstrated a dissimilarity between the amount of calories prescribed according to current nutritional guidelines and the caloric need calculated by a stress-corrected Harris–Benedict equation.	[14]
USA	Single center, comparative, longitudinal predictive study	To quantify estimation errors against indirect calorimetry measurements indirect calorimetry was used to measure resting metabolic rate for 7 days. Three estimation methods were compared with the cumulative measurement. Cumulative energy expenditure was the primary end point.	13 mechanically ventilated, ICU patients, more than 18 years old.	The actual mean 7-day cumulative difference of the sample was −618 kcal with a standard deviation of 774 kcal. This difference was equivalent to −4.7% ± 6.2% of the cumulative measured value over 7 days. The difference between measured and estimated cumulative resting metabolic rate was not statistically significant (*p* = 0.079). Among the 7 patients in group 1 (standard Penn State equation), the cumulative error of the extrapolated value compared with the measured value was −1423 ± 1524 kcal (*p* = 0.049 vs. measurement) representing −8.8% ± 9.1% of the cumulative measurement. On average, the Penn State equations predict resting metabolic rate over time within 5% of the measured value. This performance is similar to the practice of making 1 measurement and extrapolating it over 1 week.	[15]
Australia	Prospective observational study, single-center	To measure energy expenditures	20 patients within the ICU, BMI >30, >18 years old, mechanically ventilated. After enrollment, measured energy expenditure was attempted at baseline and twice weekly to extubation or day 14.	Median measured energy expenditure was 2439 (1806–2703) kcal, the study estimate was 2247 (1986–2502) kcal (or −156 (−328 to 18) kcal lower than the measured expenditure), and the guideline estimate of 11–14 kcal/kg was 1444 (1259–1500) kcal (or −950 (−1254 to −595) kcal lower than the measured expenditure). Bland–Altman bias and 95% limits of agreement between the study estimate and measured expenditure was −8% (±46%) and between the guideline estimate, −49%. Poor clinical utility; and that, furthermore, measured energy expenditure increased over time with large individual variation	[16]
**Country**	**Study Design**	**Objective of Study/Outcomes**	**Study Population/Setting**	**Effect/Consequence(s)**	**Reference**
Switzerland	Prospective interventional study over three periods (A, baseline; B and C, intervention periods). Single-center	To measure the clinical impact of a two-step interdisciplinary quality nutrition program.	572 critically ill patients, 49% on enteral nutrition (EN), 7% on EN+ parenteral nutrition (PN), Two-step quality program: (1) bottom-up implementation of feeding guideline; and (2) additional presence of an ICU dietitian.	The daily energy balance difference was significant between periods A and C with a dietitian (*p* = 0.0012), whereas it was not significant between periods A and B. The normalized daily energy delivery (kcal day ≥ 1 or kcal kg ≥ 1 day ≥ 1) improved significantly in both periods B and C. The cumulated energy balance on day 7 improved progressively over the three periods, becoming significantly less negative. The cumulated ICU stay energy balances also improved significantly. The dietitian interventions significantly improved the day 7 energy balances.	[17]
Canada	International, prospective, observational, cohort study	To develop, validate, and implement a system to reward top performers in ICU nutrition practice and to illuminate characteristics of top-performing ICUs.	81 ICUs from 18 countries. Patients: 2956 consecutively enrolled mechanically ventilated adult patients. Interventions: To qualify for the “Best of the Best” (BOB) award, sites had to have implemented a nutrition proto- col and contributed complete data on a minimum of 20 patients BOB-scoring criteria: Patients receiving enteral nutrition (EN), overall adequacy of EN plus appropriate PN, patients with EN initiated within 48 hours, patients with high gastric residual volume receiving promotility drugs or small bowel tubes, glucose measurements greater than 10 mmol/L.	The BOB award ranking ranged from 1 for the best site to 81 for the worst site. There were significant correlations between the overall BOB score and nutrition adequacy (r = 0.94). Regression analysis of the categorical variables suggested that the presence of a dietitian in the ICU was associated with a high BOB award ranking. After controlling for region, hospital size, and ICU structure, compared with ICUs without dietitians, the overall rank of ICUs with dietitians was 23.6 better.	[18]
USA	Retrospective, single-center, performance improvement project	The purpose was to evaluate the effect of registered dietitian nutritionist (RDN) order-writing privileges on enteral nutrition (EN) order compliance and nutrition delivery in ICUs.	50 critically ill patients, 150 EN days, data collection retrospectively via electronic health record.	Nonsignificant increase in EN order compliance occurred after implementation of RDN order-writing privileges, as measured by cumulative and component EN order parameters. Compliance increased by 17% for the cumulative EN order and 15% for the tube feed infusion rate order post-RDN order- writing privileges. RDN order-writing privileges improved EN order compliance and significantly improved protein delivery in selected ICUs The percent of protein needs delivered significantly increased from a mean (±SD) of 72.1% (±28.6) to 89.1% (±24.8) after implementation of RDN order- writing privileges *p* < 0.001).	[19]
Belgium	Systematic review	The review compared mainly reviews and RCTs in western countries with Nutrition Support Teams (NST) vs. a non-NST control group. Primary outcome was the prevalence of enteral nutrition vs. parenteral nutrition.	27 reviews and studies including quite heterogenic groups of patients.	There is weak evidence of that NSTs might increase appropriate EN use in ICU patients. The decrease of duration of PN could not be shown. Although almost all studies were in favor of NSTs, the evidence is weak.	[20]
USA	Single center performance improvement project	Comparison of microbial growth on different feeding product in normothermal ICU vs. hyperthermal Burn-ICU setting.	60 EN systems in normothermal (23 °C) and hyper thermal environment (32 °C), they compared open systems, “ready-to-hang”-systems (RTH) and modular, hospital-build tube feeding (MTF).	In the hyperthermal group the quantity of microbial colonization soon exceeded FDA recommendations in the MTF-group.	[21]
USA	Systematic review and meta-analysis	To assess the potential effect of methodologic bias on nutrition trials.	15 RCT, Primary (mortality, morbidity) and secondary (time on ventilator or in intensive care unit/hospital, cost) outcomes were abstracted from each trial comparing early enteral nutrition (EEN) to no/delayed enteral nutrition.	EEN had a favorable effect on mortality (RR 0.61, 95% CI 0.41, 0.89) and infectious morbidity (RR 0.80, 95% CI 0.72, 0.89), but not on non-infectious morbidity or any secondary outcome. Mortality benefit was observed only in trials with more risks of bias; infectious morbidity benefit was observed in some analyses of trials with fewer bias risks.	[22]
China	Systematic review and meta-analysis	To evaluate the efficacy and safety of enteral nutrition (EN) within 48 h after admission in the patients with severe acute pancreatitis (SAP) or predicted severe acute pancreatitis (pSAP).	10 RCT containing 1051 patients were included.	Comparing early enteral nutrition (EEN) to late EN or total parental nutrition in SAP or pSAP, the pooled risk ratios were 0.53 (95% confidence interval (CI) 0.35–0.81, *p* = 0.003) for mortality, 0.58 (95% CI 0.43–0.77, *p* = 0.0002) for multiple organ failure (MOF), 0.50 (95% CI 0.33–0.75, *p* = 0.0008) for operative intervention, 0.75 (95% CI 0.61–0.93, *p* = 0.009) for systemic infection, 0.42 (95% CI 0.26–0.69, *p* = 0.0005) for local septic complications, 0.84 (95% CI 0.74–0.96, *p* = 0.01) for gastrointestinal symptoms. 0.87 (95% CI 0.74–1.02, *p* = 0.08) for systemic inflammatory response syndrome (SIRS), and 1.24 (95% CI 0.66–2.31, *p* = 0.50) for other local complications.	[23]
Estonia	Systematic review/Delphi	To determine whether early enteral nutrition (EEN) is advisable in the heterogeneous cohort of critically ill patients and to provide evidence-based guidelines for EEN (EN started within 48 h of admission) during critical illness.	30 RCTs were analyzed. 5 meta-analyzes were performed: in unselected critically ill patients, and specifically in traumatic brain injury, severe acute pancreatitis, gastrointestinal (GI) surgery and abdominal trauma.	EEN reduced infectious complications in unselected critically ill patients, in patients with severe acute pancreatitis, and after GI surgery. Did not detect any evidence of superiority for early parenteral nutrition or delayed EN over EEN.	[24]
Australia	Meta-analysis	To determine whether the provision of early standard enteral nutrition (EN) confers treatment benefits to adult trauma patients who require intensive care.	RCTs conducted in adult trauma patients requiring intensive care that compared the delivery of standard EN, provided within 24 h of injury, to standard care were included. Outcomes included mortality, functional status and quality of life. Secondary analyses considered vomiting/regurgitation, pneumonia, bacteriaemia, sepsis and multiple organ dysfunction syndrome.	Three RCTs with 126 participants were found to be free from major flaws and were included in the primary analysis. The provision of early EN was associated with a significant reduction in mortality (OR = 0.20, 95% confidence interval 0.04–0.91, I2 = 0). No other outcomes could be pooled.	[25]
USA	RCT, single-center	Comparing early trophic enteral nutrition (EN) with “no EN” in mechanically ventilated adults with septic shock	31 patients, adults who were at least 18 years of age, admitted to the medical ICU with a primary diagnosis of septic shock, and mechanically ventilated within 24 h of ICU admission.	31 patients, 15 received early EN, 16 randomized to receive no EN. Early trophic EN group started EN with a 1.2-kcal/mL formula within 16 hours (interquartile range (IQR) 9–21) from ICU admission, and the “no EN” group started EN 48 hours (IQR 13–61) after ICU admission. Twenty percent of early EN patients had a vomiting episode over the first 7 days, as compared with 56% in the “no EN” group (*p* = 0.038). No patient had bowel obstruction, or ileus. One patient in the “no EN” group had VAP, compared with 0 in the early trophic EN group. Candida was isolated in subsequent urine or respiratory culture in 6/16 (38%) patients in the “no EN” group and in 1/15 (7%) patient from the early EN group (*p* = 0.083).	[26]
China	Systematic review and meta-analysis	To evaluate the effect of early enteral nutrition (EEN) on the outcome of critically ill patients.	1725 patients, RCTs conducted in critically ill patients that compared the EEN, provided within 48 h of ICU admission or post-operation, to delayed enteral nutrition (DEN) were included.	Although no significant difference was observed in the risk of mortality, EEN within 48 h can improve the clinical outcomes of critically ill patients compared to DEN. This study showed that EEN within 48 h of admission is associated with a reduced risk of complications, infection, pneumonia, and length of stay compared to DEN.	[27]
Australia	Meta-Analysis of Randomized Controlled Trials	To identify, appraise, and synthesize the most current evidence to determine whether early enteral nutrition (EN) alters patient outcomes from critical illness.	699 full-text articles were retrieved and screened. Sixteen randomized controlled trials enrolling 3225 critically ill participants were included.	Compared with all other types of nutrition support, commencing EN within 24 hours of ICU admission did not result in a reduction in mortality (odds ratio, 1.01; 95% CI, 0.86–1.18; *p* = 0.91; I2 = 32%). However, there was a differential treatment effect between a priori identified subgroups (*p* = 0.032): early EN reduced mortality compared with delayed enteral intake (odds ratio, 0.45; 95% CI, 0.21–0.95; *p* = 0.038; I2 = 0%), whereas a mortality difference was not detected between early EN and PN (odds ratio, 1.04; 95% CI, 0.89–1.22; *p* = 0.58; I2 = 30%). Overall, patients who were randomized to receive early EN were less likely to develop pneumonia (odds ratio, 0.75; 95% CI, 0.60–0.94; *p* = 0.012; I2 = 48%).	[28]
**Country**	**Study Design**	**Objective of Study/Outcomes**	**Study Population/Setting**	**Results/Consequence(s)**	**Reference**
Australia	Prospective before and after study, single-center study	Number of interruptions and the reasons for interrupting, development of recommendations for nursing practice	Before (338 patients), after (315 patients) Development of strategies to improve practice and minimize the effect to practice.	Number of interruptions decreased from 907 to 662. Interruptions due to gastrointestinal issues decreased (14 vs. 10%). Time lost to feeding because of interruptions was similar in both groups.	[29]
Japan	Single-center retrospective chart review	Duration of interruption; reason for each interruption, presence of written orders for interruptions.	Retrospective chart review of 100 patients	There were 567 episodes of enteral nutrition (EN) interruption over a median ICU length of stay of 17.1 days. There were a median of three EN interruption episodes per patient. Median duration of EN interruption in all patients was 5.5 h.	[30]
Malaysia	Prospective observational study, single-center	Prevalence, causes, and duration of such interruption were investigated.	154 ICU patients	About 332 episodes of interruptions were recorded. For each patient, feeding interruptions occurred for a median of 3 days. Total duration of feeding interruptions for the entire ICU stay: 24.5 h. Median energy and protein deficits per patient due to feeding interruptions for the entire ICU stay were 1780.23 kcal and 100.58 g.	[31]
Canada	Retrospective review	To examine differences between prescribed and actual enteral nutrition (EN) delivery and to identify the specific causes of EN interruption and to quantify these.	Adult regional American Burn Association-verified burn center, total of 90 subjects were studied. On postburn days 0 to 14 the daily volume of EN prescribed by the dietitian was compared with the actual volume received by the patient.	Enterally fed burn patients received significantly less nutrition than prescribed. Interruptions for surgery accounted for 24% of total discrepancy time. Other causes of discrepancies were physician- or nurse-directed interruptions (16% of time), planned extubation (7%), feed intolerance (11%), tube malfunction (2%), bedside procedures (2%), and dressing changes (3%).	[32]
UK	A service improvement project	To evaluate the effectiveness of a fasting guideline in a general/trauma ICU.	A general/trauma ICU in a London teaching hospital. The unit takes approximately 700 admissions a year, with 30–50% of admissions being trauma. There are eight intensive care medicine consultants, 100 nursing staff (50% band 5, 38% band 6, 10% band 7 and 2% band 8) and full-time critical care specialist dietitian, pharmacist and physiotherapists.	There were 62 interruptions to enteral nutrition delivery with the first data collection and 64 in the second. Prolonged fasting before and after surgery and airway procedures were initially identified as the two most important causes of delays.	[33]
Iran	RCT, single center	Comparison of gastric residual volume (GRV) by position (supine, semirecumbent (SR), and right lateral (RL)) and by group (A and B). Groups A and B were in the supine position in Stage 1. Group A was in the SR position in Stage 2 and in the RL position in Stage 3. Group B was in the RL position in Stage 2 and in the SR position in Stage 3.	36 mechanically ventilated patients. GRV was measured 3 h after feeding in supine and then in right-lateral and semi-recumbent position.	No significant difference in the GRV between groups while in the supine position (*p* = 0.085), SR position (*p* = 0.106), or RL position (*p* = 0.059). The effect of group (A vs. B) and position (supine, SR, or RL) on GRV was statistically significant for both groups (both at *p* = 0.001). GRV was significantly lower in the SR position compared with the supine position in both groups (*p* < 0.05), and GRV in the RL position was significantly lower than in the supine position in both groups (*p* < 0.05). GRVs in the SR and RL positions, although significantly and respectively different from the supine position, were not significantly different from each other (*p* > 0.05).	[34]
Brazil	Systematic review	To evaluate the effect of enteral feeding of critically ill adult and pediatric patients in the prone position on gastric residual volume and other clinical outcomes.	Four studies with adult patients and one with preterm patients were included. Main outcome = gastric residual volume	Three studies did not show differences in the gastric residual volume between the prone and supine positions (*p* > 0.05), while one study showed a higher gastric residual volume during enteral feeding in the prone position and another group observed a greater gastric residual volume in the supine position (reduction of the gastric residual volume by 23.3% in the supine position versus 43.9% in the prone position; *p* < 0.01). Two studies evaluated the frequency of vomiting; one found that it was higher in the prone position (30 versus 26 episodes; *p* < 0.001), the other study no significant difference (*p* > 0.05).	[35]
France	Before–after study, single-center	To evaluate an intervention for improving the delivery of early enteral nutrition (EEN) in patients receiving mechanical ventilation with prone positioning (PP).	Eligible patients receiving EEN and mechanical ventilation in PP were included within 48 h after intubation in a before–after study. Patients were semi-recumbent when supine.	An intervention including PP with 25° elevation, an increased acceleration to target rate of EN, and erythromycin improved EN delivery. Compared to the before group, larger feeding volumes were delivered in the intervention group (median volume per day with PP, 774 ml (IQR 513–925) vs. 1170 mL (IQR 736–1417); *p* < 0.001) without increases in residual gastric volume, vomiting, or ventilator-associated pneumonia.	[36]
France	RCT, multicenter, open-label, parallel-group study	To evaluate the outcome of early first-line enteral nutrition versus parenteral nutrition	2410 patients on 44 ICU	Higher rate of digestive complication in enteral nutrition. Vomiting (406 (34%) vs. 246 (20%)); HR 1.89 ((1.62–2.20); *p* < 0.0001), diarrhoea (432 (36%) vs. 393 (33%)); 1.20 ((1.05–1.37); *p* = 0.009), bowel ischaemia (19 (2%) vs. five (<1%)); 3.84 ((1.43–10.3); *p* = 0.007), and acute colonic pseudo-obstruction (11 (1%) vs. three (<1%)); 3.7 ((1.03–13.2); *p* = 0.04).	[37]
Saudi Arabia	Prospective, randomized, multi-center study	Primary outcome was 90-day all-cause mortality.	894 ICU patients. Randomly assigned to either permissive underfeeding or standard enteral feeding group.	No significant difference of 90-day mortality between the groups. Of patients in the standard enteral feeding group, 17.7% developed feeding intolerance, which summarizes vomiting, abdominal distention, or a gastric residual volume of more than 200 mL.	[38]
Turkey	Prospective observational study, single-center study	To evaluate the frequency, risk factors and complications of gastrointestinal dysfunction during enteral nutrition (EN) in the first 2 weeks of the ICU stay and to identify precautions to prevent the development of gastrointestinal dysfunction and avoid complications.	137 ICU patients	Gastrointestinal dysfunction can cause inadequate nutrition. Incidence of gastrointestinal dysfunction was 63% (diarrhea 26%, constipation 29%, upper digestive intolerance 36%, vomiting 19%). Negative fluid balance and MDR bacteria positivity were independent risk factors for gastrointestinal dysfunction.	[39]
Switzerland	Prospective double-blind, RCT, single-center pilot study	Assessment of incidence and frequency of diarrhea and the respective effects of a modified enteral diet compared to a standard diet.	90 ICU patients with enteral tube feeding. 1:1 randomization, receiving either a standard formula or Peptamen^®^ AF, rich in proteins, medium chain triglycerides and fish oil. (intervention, *n* = 46; control, *n* = 44).	The incidence of diarrhea was 64% in the intervention and 70% in the control group. Diarrhea was associated with length of mechanical ventilation (9.5 (6.0–13.1) vs. 3.9 (3.2–4.6) days; *p* = 0.006) and length of ICU stay (11.0 (8.9–13.1) vs. 5.0 (3.8–6.2) days; *p* = 0.001)	[40]
UK	Retrospective, multi-center study	Prevalence, risk factors, clinical consequences, and treatment of enteral feed intolerance on ICU.	1888 patients, 167 ICU’s	Incidence of intolerance was 30.5% after a median 3 days from enteral nutrition (EN) initiation and led to prolonged ventilation (2.5 vs. 11.2, *p* < 0.0001), increased ICU stay (14.4 vs. 11.3 days, *p* < 0.0001), and increased mortality (30.8% vs. 26.2, *p* = 0.04).	[41]
Malaysia	Prospective, observational study, single-center study	Incidence, risk factors, and outcome of refeeding syndrome.	109 ICU patients	42.6% developed refeeding hypophosphatemia.	[42]
Turkey	Retrospective, single-center study	Incidence of refeeding hypophosphatemia.	117 ICU patients with enteral nutrition (EN), parenteral nutrition (PE) or EN + PN	Overall incidence was 52.14%. Refeeding hypophosphatemia was found in 47.5% of the patients with PN, in 55.17% of the patients with EN, and in 52.6% of the patients with EN + PN. Mortality rate was significantly higher in patients with hypophosphatemia than without. (*p* = 0.037).	[43]
USA	Retrospective, case-control, single-center study	Incidence of enteral nutrition (EN) induced hypophosphatemia	213 patients, surgical ICU	59% incidence of hypophosphatemia of any cause. 33% refeeding hypophosphatemia 39% non-refeeding hypophosphatemia Not associated with worse clinical outcome.	[44]
UK	Systematic review and meta-analysis	Effectiveness of nasogastric versus post-pylorus feeding in ICU.	20 RCT’s 1496 patients	Lower rate of aspiration pneumonia in post-pyloric feeding (nasojejunal tube) (OR, 1.41; 95% CI, 1.01, 1.98; z = 2.03; *p* < 0.04; I-2 = 10%)	[45]
Italy	Retrospective cohort study	To compare the occurrence of diarrhea in patients fed with blenderized natural food diet compared to commercial enteral feeding preparation.	215 patients on cardiac-surgery ICU.	Commercial enteral feeding was administered by continuous pump infusion. (*n* = 112). Natural enteral feeding was administered by bolus 3 times per day (*n* = 103). Development of diarrhea was significantly lower in the blenderized natural food diet group compared to the commercial enteral feeding preparation group. (27.2% versus 48.2%, *p* = 0.002)	[46]
Canada	Systematic review and meta-analysis	To evaluate the effectiveness and safety of post-pyloric feeding versus gastric feeding for critically ill adults who require enteral tube feeding.	14 eligible studies including 1109 ICU patients	There was no difference in mortality or duration of mechanical ventilation between the groups. Post-pyloric feeding is associated with lower rates of pneumonia compared with gastric tube feeding. (moderate quality of evidence; RR 0.65, 95% confidence interval (CI) 0.51 to 0.84.	[47]
Israel	Guideline	ESPEN Guideline “Clinical Nutrition on intensive care unit”	Literature review and Expert opinion.	Possible complications as well as energy and protein deficit.	[1]
UK	Retrospective single center study	To study the effect of implementation of fasting guideline	74 ICU patients	77% of staff were familiar with the guidelines, whilst 42% requested further education. The main barriers to guideline compliance were delays and unpredictable timing of procedures, and differing guidance from senior staff and non-ICU teams. Significant improvement in enteral nutrition (EN) delivery and reduced duration of feed breaks when using a protocol.	[48]
Germany	Before and after design, single-center study	To examine whether early enteral nutrition (EN) of critically ill patients could be improved by a nurse-driven implementation of an existing feeding protocol.	A total of 101 and 97 patients were included, respectively, before and after the intervention.	Following intervention, EN started significantly earlier (28 ± 20 h versus 47 ± 34 h, *p* < 0.001), within 24 h in 64% versus 25% (*p* < 0.0001). For each of the first 5 days, the proportion of patients meeting their nutritional goal was significantly higher.	[49]
Canada	International, prospective, observational, cohort studies, multicenter study	To evaluate the effect of enteral feeding protocols on key indicators of enteral nutrition in the critical care setting.	5497 consecutively enrolled, mechanically ventilated, adult patients, 269 intensive care units (ICUs) in 28 countries.	Protocolized sites used more enteral nutrition (EN) alone (70.4% of patients vs. 63.6%, *p* = 0.0036), started EN earlier (41.2 hours from admission to ICU vs. 57.1, *p* = 0.0003), and used more motility agents in patients with high gastric residual volumes (64.3% of patients vs. 49.0%, *p* = 0.0028) compared with sites that did not use a feeding protocol. Overall nutritional adequacy (61.2% of patients’ caloric requirements vs. 51.7%, *p* = 0.0003) and adequacy from EN were higher in protocolized sites compared with non-protocolized sites (45.4% of requirements vs. 34.7%, *p* < 0.0001).	[18]
China	Prospective multi-center before-after study, conducted in 15 ICU’s of general hospitals in China	To explore the effects of an enteral nutrition (EN) feeding protocol (simplified-five-step SFS) in critically ill patients. Primary endpoint was the percentage of patients receiving EN within 7 days after ICU admission.	439 (209 control, 230 intervention) patients in ICU for more than 3 d	SFS implementation did not increase percentage of patients receiving EN within 7 days (*p* = 0.65). EN related Adverse Events: 31.5 of 1000 ICU patient days in the control group and 19.1 of 1000 in the intervention group with statistical difference (*p* = 0.004). EN feeding protocol might be associated with increase of hospital survival (OR: 0.74, 95% CI: 0.55–1.00, *p* = 0.046) Intervention group reached significantly higher percentage of estimated calorie targets on day 6 (*p* = 0.01) and 7 (*p* = 0.002) than control.	[50]
Brazil	Single-center prospective cohort study	To determine how often daily calorie goals are met and the factors responsible for inadequate nutrition support.	262 daily evaluations in 40 patients	Most patients did not achieve the prescribed daily calorie goal→ associated with the use of midazolam and assistance by a reduced nursing staff. Daily calorie goal was achieved in only 46.2% of the evaluations (*n* = 121) Risk factors for inadequate nutrition support were the use of midazolam (odds ratio, 1.58; 95% CI, 1.18–2.11) and fewer nursing professionals per bed (odds ratio, 2.56; 95% CI, 1.43–4.57).	[51]
Jordan	Multicenter Survey Study	To explore Jordanian ICU nurses’ perceived barriers for enteral nutrition that hinders them from utilizing the recommended enteral nutrition (EN) guidelines.	131 ICU nurses	Most patients did not achieve the prescribed daily calorie goal, associated with the use of midazolam and assistance by a reduced nursing staff. The most important barrier was “Not enough nursing staff to deliver adequate nutrition” (M = 4.80, SD = 1.81, 60%), followed by “Fear of adverse events due to aggressively feeding patients” (M = 4.59, SD = 1.50, 56%).	[52]
USA	Cross-sectional survey, multicenter study	To describe the barriers to enterally feeding critically ill patients from a nursing perspective and to examine whether these barriers differ across centers.	A total of 138 of 340 critical care nurses completed the questionnaire.	No or not enough dietitian coverage during weekends and holidays. The 5 most important barriers to nurses were (1) other aspects of patient care taking priority over nutrition, (2) not enough feeding pumps available, (3) enteral formula not available on the unit, (4) difficulties in obtaining small bowel access in patients not tolerating enteral nutrition, and (5) no or not enough dietitian coverage during weekends and holidays.	[53]
China	Cross-sectional descriptive multicenter study	To investigate the barriers in administering enteral feeding to critically ill patients from the nursing perspective.	808 nurses from 10 comprehensive hospitals	Frequency of enteral nutrition (EN)-related training, full-time ICU nutritionist, hospital level, specific protocols for enteral feeding and position were significantly influencing the enteral feeding of ICU patients.	[54]
USA	Prospective single-center observational pilot study	Nutrition education program would improve our residents’ knowledge of ICU nutrition.	8 surgery residents completed the nutrition education program. Pre- and post-testing were performed to assess short-term comprehension.	The nutrition education program improved both short-term and long-term ICU nutrition knowledge of surgery residents (*p* < 0.01).	[55]
Australia	Online questionnaire	To explore Australian nurses’ enteral nutrition (EN) knowledge and sources of information.	359 responses of registered nurses	Most respondents reported their EN knowledge was good (*n* = 205, 60.1%) or excellent (*n* = 35, 10.3%), but many lacked knowledge regarding the effect of malnutrition on patient outcomes. Dietitians and hospital protocols were the most valuable sources of enteral nutrition information, but were not consistently utilized.	[56]
South Korea	Quasi-experimental, one-group study with a pre- and post-test design, multicenter study	To evaluate the effects of an education program to improve critical care nurses’ perceptions, knowledge, and practices towards providing enteral nutritional support for ICU patients.	Nurses (*n* = 205) were recruited from nine ICUs.	Nurses’ overall perception significantly improved after the program (mean change = 3.18, *p* < 0.001). Nurses’ knowledge about enteral nutritional support showed a significant improvement after the education program (mean change = 11.2%, *p* < 0.001). Nurses’ total practice score significantly improved after the program (mean change = 2.54, *p* < 0.001).	[57]
Brazil	Prospective, non-blinded single-center study	To evaluate the impact of a multifaceted nutritional educational intervention on the quality of nutritional therapy and clinical outcomes in critically ill patients.	16-bed ICU Phase 1: the quality of NT was evaluated in 50 newly admitted ICU patients in a pre-educational program (Pre-EP). Phase 2: nutritional protocols were created and an education program was implemented. Phase 3: another 50 patients were enrolled and observed in a post-educational program (Post-EP) using phase.	The mean ± SD duration of fasting decreased (Pre-EP 3.8 ± 3.1 days vs. Post-EP: 2.2 ± 2.6 days; *p* = 0.002), the adequacy of nutritional therapy improved (Pre-EP 74.2% ± 33.3% vs. Post-EP 96.2% ± 23.8%; *p* < 0.001), and enteral nutrition was initiated earlier than 48 h more commonly (Pre-EP 24% vs. Post-E 60%; *p* = 0.001). Median ICU length of stay decreased (Pre-EP: 18.5 days vs. Post-EP: 9.5 days; *p* < 0.001) although hospital length of stay did not.	[58]
UK	Prospective, pre- and post-intervention single-center study	To assess accuracy of enteral feeding records, to increase nursing education and to improve nutritional documentation.	188 patient electronic medical records (EMR)	The intervention of an education program reduced the documented discrepancy between the pump readings and charted volumes from 44 to 33%. A correlation analysis also showed a tighter relationship post-intervention (rpost = 0.84 vs. rpre = 0.76, both had a *p* < 0.01).	[59]
USA	Prospective clinical trial	The experimental group (EG) received targeted education consisting of strategies to increase delivery of early enteral nutrition. Strategies included early enteral access, avoidance of nil per os (NPO) and clear liquid diets (CLD), volume-based feeding, early resumption of feeds post procedure, and charting caloric deficits. The control group (CG) did not receive targeted education but was allowed to practice in a standard ad hoc fashion.	Patients (*n* = 121) assigned to 1 of 2 trauma groups	EG received a higher percentage of measured goal calories (30.1 ± 18.5%, 22.1 ± 23.7%, *p* = 0.024) compared with the CG. Mean caloric deficit was not significantly different between groups (−6796 ± 4164 kcal vs. −8817 ± 7087 kcal, *p* = 0.305). CLD days per patient (0.1 ± 0.5 vs. 0.6 ± 0.9), length of stay in the intensive care unit (3.5 ± 5.5 vs. 5.2 ± 6.8 days), and duration of mechanical ventilation (1.6 ± 3.7 vs. 2.8 ± 5.0 days) were all reduced in the EG compared with the CG (*p* < 0.05). EG patients had fewer nosocomial infections (10.6% vs. 23.6%) and less organ failure (10.6% vs. 18.2%) than did the CG, but these differences did not reach statistical significance.	[60]
Canada	Questionnaire Multicenter Evaluation, enquiry study	This study describes the results of an evaluation of educational strategies used to implement a novel enteral feeding protocol.	The response rate to the questionnaire was 166 of 434 or 38.2%.	More than 70% of respondents rated 5 of the educational strategies as very useful or somewhat useful. The percentage of nurses who found the bedside protocol tools of the enteral feeding order set, gastric feeding flowchart, and volume-based feeding schedule either “very easy” or “somewhat easy” to use were 64.0%, 60.5%, and 59.1%, respectively.	[61]
USA	Creation of a Web-Based Teaching Module	The authors created a self-directed Web-based teaching module (WBTM) to educate and standardize placement of postpyloric nasoenteric tube (NET).	Forty-three first-, second-, or third-year residents or medical or physician assistant students took pretests for knowledge and confidence surveys, viewed the WBTM, placed NET at the bedside, then took a posttest and confidence survey while awaiting confirmation of tube position by abdominal radiograph.	Knowledge and confidence significantly improved. Overall success rate of postpyloric NET placement for all participants on first attempt was 74.4% vs. 46.7% in the control (*p* = 0.005). Improvement occurred in all subgroups, including those with no prior experience, who were successful 70.4% of the time (*p* = 0.009).	[62]

## Data Availability

The datasets used and/or analysed during the current study are available from the corresponding author on reasonable request.

## Supplementary Materials

The following are available online at https://www.mdpi.com/2072-6643/13/1/82/s1, Table S1: Risk-Checklist.

## Abbreviations

ASPEN	American Society for Parenteral and Enteral Nutrition
CIRS	Critical Incident Reporting System
DIN EN ISO 80369-3	Deutsches Institut für Normung and designation of the standard
e.g.,	exempli gratia: for example
EN	Enteral Nutrition
ENFit™-technology	Connector for safe feeding
ESPEN	European Society for Clinical Nutrition and Metabolism
GI	gastrointestinal
i.e.,	id est: that is
ICU	Intensive Care Unit
MeSH	Medical Subject Headings
MNA	Mini Nutritional Assessment
mNUTRIC	modified NUTRIC score
MTF	modular hospital-built tube feeding systems
MUST	Malnutrition Universal Screening Tool (MUST)
NRS-2002	Nutrition Risk Screening 2002
PN	Parenteral Nutrition
RCTs	Randomized Controlled Trials
RHT systems	“ready-to-hang”-systems
SAG	Subjective Global Assessment
SOPs	Standard Operating Procedures (SOPs)
VCO2	volume of carbon dioxide
VO2	volume of oxygen
