# Supplementary material for: Risks in Management of Enteral Nutrition in Intensive Care Units: A Literature Review and Narrative Synthesis
Source: Nutrients. 2020 Dec 29;13(1):82. doi: 10.3390/nu13010082 (PMC7823864; doi:10.3390/nu13010082)
Supplement: Supplementary file 1 [file nutrients-13-00082-s001.pdf]

Risk-Checklist

Identifying risks in management of enteral nutrition in intensive care units (ICU)

ICU Name :____________________ Date: ______________________________

Rate from low (1) to high (6) risk	1	2	3	4	5	6	Comments
**Admission**							
1. No use of clinical assessment or screening nutrition assessment	☐	☐	☐	☐	☐	☐	
2. Inadequate tube management and position	☐	☐	☐	☐	☐	☐	
**Prescribing**	☐	☐	☐	☐	☐	☐	
3. Missing energy target	☐	☐	☐	☐	☐	☐	
**Verifying**	☐	☐	☐	☐	☐	☐	
4. Missing a nutritionist at the ICU	☐	☐	☐	☐	☐	☐	
**Preparing**	☐	☐	☐	☐	☐	☐	
5. Insufficient hygiene and handling	☐	☐	☐	☐	☐	☐	
**Administering**	☐	☐	☐	☐	☐	☐	
6. Wrong time management, speed and route	☐	☐	☐	☐	☐	☐	
7. Nutritional interruptions	☐	☐	☐	☐	☐	☐	
8. Wrong body position	☐	☐	☐	☐	☐	☐	
**Monitoring**	☐	☐	☐	☐	☐	☐	
9. Gastrointestinal complication and infections	☐	☐	☐	☐	☐	☐	
**General risks**	☐	☐	☐	☐	☐	☐	
10. Missing or not using guidelines, standards or protocols	☐	☐	☐	☐	☐	☐	
11. Understaffing	☐	☐	☐	☐	☐	☐	
12. Lack of education	☐	☐	☐	☐	☐	☐	

7-Step-Instruction

1. Please insert name of ICU and date
2. Use checklist within different experts of different professions (physician, nurse, dietician, etc.) with different level of work experience
3. Compare results and discuss
4. Set actions, if necessary
5. Evaluate your actions by using this checklist again
6. Compare results and discuss
7. Take measures, where necessary

Author: Magdalena Hoffmann (2020) - Risk Checklist

ICU Name :___________________	Date: ______________________________						
**Rate from low (1) to high (6) risk**	**1**	**2**	**3**	**4**	**5**	**6**	**Comments**
**Insert additional risks such as:**							
Nosocomial infection							
Medication error							
Overseen allergies							
Insufficient pain management							
Communication failures							
Documentation failures							
Failure in handling of medical devices							
Incorrect patient identification							
…							

7-Step-Instruction
